# Correspondence

**Published:** 1899-01

**Authors:** 


### CORRESPONDENCE.

KANKAKEE, Ill., December 15, 1898.

TO THE MEMBERS OF THE PROFESSION IN ILLINOIS:

GENTLEMEN: At the last annual meeting of the Illinois State Veterinary Medical Association, held in Chicago, November 16 and 17, 1898, it was decided to again take such action as would secure the passage of a law regulating the practice of veterinary medicine and surgery in this State. With this object in view it was decided to call on each and every member of the veterinary profession in this State, to ask of the Senator and Representative of his district, either by a personal interview or by letter, to give their active aid and support to secure the passage of a just and equitable law that will place the members of the veterinary profession in this State on an equal basis with those of the other learned professions.

The next meeting of the State Association will be held at the Leland Hotel, in Springfield, February 15, 1899. All qualified members of the profession in this State, whether members of the Association or not, are cordially invited to attend this meeting and take part in the discussion concerning the best methods of elevating the standard of our calling.

W. J. MARTIN,
President Illinois State Veterinary Medical Association.

### AS TO STATE VETERINARIAN OF PENNSYLVANIA.

HARRISBURG, PA., January 5, 1899.

EDITORS JOURNAL OF COMPARATIVE MEDICINE, ETC.

I HAVE recently had occasion to refer to the growth of the veterinary sanitary service of Pennsylvania, and stated that prior to 1895 there was no State Veterinarian.

My attention has been called to the fact that an impression exists that the office mentioned is not of such recent origin. This impression is no doubt based on the assumption that the offices of Veterinary Surgeon to the State Board of Agriculture and State Veterinarian are the same.

That such is not the case is shown by the fact that both offices exist to-day, and the duties of their incumbents do not conflict or overlap in any way.

LEONARD PEARSON.

### SHALL THE VETERINARIAN PERFORM THE OPERATION OF DOCKING FOR FASHION?

EDITORS JOURNAL OF COMPARATIVE MEDICINE, ETC.

THE Royal Agricultural Society and the Hunters' Improvement Society, the principal horse-show societies of England, recently passed a resolution providing that "in 1899 no docked yearling is eligible for any show held under the rules of the above societies; in 1900 no two-year-old that has been docked can be shown; in 1901 no three-year-old," and so on, until no more docked horses will be shown. It is well known that the only reason that docked tails ever appeared in this country was because it was the fashion in England. And as it would seem from the above that English aristocracy have now become tired of it, and the horse-show societies are discouraging the practice, it is to be hoped that we Americans will imitate the English in their good sense as thoroughly as we have in their folly, and that steps will be taken through sources of influence to abolish it here.

One source that can accomplish much in this respect is the veterinarian individually, and collectively as veterinary societies. There is some difference of opinion, however, even among veterinarians upon this subject, and in discussions upon it we often hear it argued that if the people demand horses with their tails docked they will have them, and the veterinarian is the one to do the operation; therefore he should not consult his own opinion in the matter. In all professions conditions are met with that rise paramount to pecuniary considerations, and if the refusal of the veterinarian to perform an operation will assist in the abolishment of a revolting cruelty to a helpless brute, it would seem that, as the professed protector and healer of his troubles, the dumb animal had some claims upon him for that assistance. But, aside from humane considerations, I think any one taking the trouble to investigate will find that the demand for short tails is not as prevalent as supposed, even in our Eastern cities.

The buyers of coach horses and so-called horses of fashion, as a rule, are careless of the matter, but there is yet sufficient demand among those who will have no other to cause this class of horses to be placed upon the market, generally with docked tails.

The number of docked horses seen upon our streets is not a true indicator of the degree of its popularity, for it should not be supposed that every owner of a tailless horse is in favor of the practice. There are many purchasers of the heavier carriage type of horse who would prefer him with his tail; but when such a buyer goes into the market he finds that docked tails largely predominate in this class of horses, and he probably accepts the one conforming to his ideal in other respects. This is so because dealers realize that stump-tails are still considered fashionable, and their business is one of revenue, not sentiment. But the cause for this is the same that would cause a demand for horses with every hair shaved from the tail if fashion so decreed. It is a demand from a class of people who care nothing for the horse individually, and one which has not a sound argument in support of its existence. Laws against the practice will not stop it any more than prohibiting the saloon will stop the traffic in liquor. As long as demand exists supply will be forthcoming, and this must be borne in mind by those who would effectually oppose it. It is an acquired morbidness of taste that fosters such insults to nature, and it is this that must be contended with. Certainly no sane person can think that a docked tail adds to a horse's beauty. The anatomical scenery so persistently held up for the inspection of the driver of such a poor, unfortunate beast is hardly one to inspire joy, especially if a lady companion is sharing the pleasures of the drive.

I believe that a large majority of veterinarians are averse to the practice of docking, and certainly they are a class who can wield a powerful influence toward its abolishment in this country by their intelligently directed efforts. In times past it has fallen to their lot to stamp out contagious diseases of foreign origin among our livestock. Let them now bring the power that is theirs to bear upon a public opinion that will eventually snuff out this invasion of toadyism that affects admiration for this disgusting vandalism upon the most beautifully symmetrical of all animals—one placed in the care of man for his development and companionship, and thus mutilated. Why? Because it is a practice sanctioned by those horse-owners who have no thought or special choice in the matter, and is maintained by a class upon whom the burden rests of inaugurating it in this country—a class who will imitate the freaks of an aristocracy foreign to all principles of Americanism, servants to that which in men and women we call fashion, but which in the monkey is called aping.

B. M. Underhill, V.M.D.,
Media, Pa.

---

The smallest camels are native to Persia. They are not more than twenty inches high.

